# Targeting the STAT5 pathway in Ph+ acute lymphoblastic leukemia

**DOI:** 10.18632/oncotarget.26412

**Published:** 2018-12-04

**Authors:** Valentina Minieri, Marco De Dominici, Marja T. Nevalainen, Bruno Calabretta

**Affiliations:** Bruno Calabretta: Department of Cancer Biology, Sidney Kimmel Cancer Center, Thomas Jefferson University, Philadelphia, PA, USA

**Keywords:** oncogene, apoptosis, leukemia, transcription factor, signal transduction

**Figure 1:**
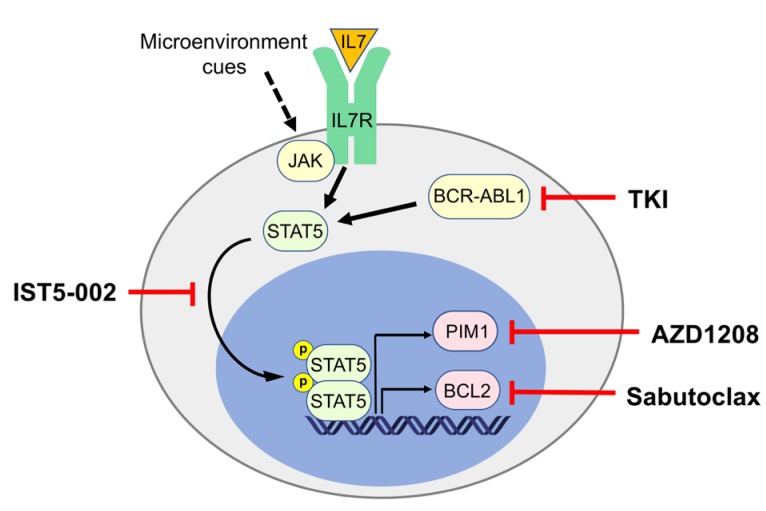
STAT5 activation in Ph+ ALL and pathway inhibitors STAT5 is phosphorylated and activated in Ph+ ALL cells by means of JAK-dependent microenvironmental cues, including activation of the IL-7 receptor, and, most importantly, by BCR-ABL1, directly or *via* JAK2. STAT5 activity can be prevented by inhibiting its phosphorylation by BCR-ABL1, by using direct STAT5 inhibitors such as IST5-002 or by acting on its transcriptional targets such as PIM-1 and BCL-2 proteins.

Ph+ ALL accounts for up to 25-30% of adult ALL, and its clinical outcome remains unfavorable in approximately 50% of the patients despite the introduction of more effective induction and consolidation therapies consisting of BCR-ABL1 tyrosine kinase inhibitors (TKI) in combination with cytotoxic agents followed by hematopoietic stem cell transplantation (HSCT) [1]. Resistance to TKIs develops in most patients through the outgrowth of leukemic subclones carrying mutations in the BCR-ABL1 kinase domain that impede the binding of these drugs to the ATP-binding pocket. Less frequently, resistance develops in the absence of tyrosine kinase domain mutations, implying that BCR-ABL1-independent mechanisms may be crucial in such instances. In particular, such mechanisms may involve microenvironment-regulated pathways that bypass BCR-ABL1-dependent cell-autonomous growth-promoting signals. Hence, a more effective therapy of Ph+ ALL, especially at the TKI-resistant stage, is likely to depend on targeting growth-promoting pathways that are simultaneously activated *via* cell-autonomous and microenvironment-dependent mechanisms. One such pathway is STAT5 that is constitutively active in Ph+ ALL cells through BCR-ABL1 and can be activated by JAK2 *via* stroma-derived cytokines (Figure 1).

The role of STAT5 for maintenance of oncogenic ABL1-driven ALL has been previously investigated in STAT5-deficient transgenic mice [2, 3]. Although disease outcomes were, in part, influenced by the genetic background of recipient mice, such studies supported the relevance of STAT5 in growth and progression of BCR-ABL1-driven leukemias. However, there is little information on the requirement of STAT5 and STAT5-regulated pathways in patient-derived Ph+ ALL and whether such pathways can be targeted therapeutically in xenograft models of human Ph+ ALL.

In a recently published study [4], we used genetic and pharmacological approaches to investigate the consequences of inhibiting the activity of STAT5 and STAT5-regulated pathways for *ex vivo* growth and leukemogenesis of Ph+ cell lines and patient-derived newly diagnosed and relapsed/TKI-resistant Ph+ ALL.

Compared to controls, Ph+ ALL cell lines and primary Ph+ ALL cells lentivirally transduced with a STAT5 shRNA or treated with IST5-002, a recently developed small molecule STAT5 inhibitor [5], exhibited reduced cell growth and enhanced apoptosis. IST5-002 has been shown to inhibit phosphorylation, dimerization, nuclear translocation, DNA binding and transcriptional activity of STAT5 [5]. In addition, doxycycline-induced activation of a STAT5 shRNA or treatment with IST5-002 led to a markedly increased survival of immunodeficient mice injected with Ph+ ALL cell lines or Ph+ ALL cells derived from a patient with TKI-resistant disease.

Induction of apoptosis in STAT5-silenced Ph+ ALL cells was associated with downregulation of BCL-2 and MCL-1 and upregulation of BIM expression. In STAT5-silenced Ph+ BV173 cells, apoptosis was rescued by BIM silencing or by ectopic BCL-2 expression while restoration of MCL-1 levels had no effects on cell viability. However, it is possible that other Ph+ ALL cell lines and, more importantly, primary Ph+ ALL cells rely on MCL-1 expression for their survival.

Inhibition of cell growth induced by STAT5 silencing in Ph+ ALL cells was also associated with decreased expression of c-MYC and the PIM-1 kinase. Surprisingly, restoring PIM-1 expression in STAT5-silenced Ph+ ALL cells did not rescue growth inhibition induced by STAT5 silencing. This is most likely due to the inability of PIM-1 to suppress the apoptosis of STAT5-silenced Ph+ ALL cells which is associated with upregulation of BIM levels. In contrast, pharmacological inhibition of PIM kinase by treatment with the PIM1/2 kinase inhibitor, AZD1208, suppressed proliferation and colony formation of Ph+ ALL cells, including primary leukemia cells from newly diagnosed and relapsed patients. AZD1208 suppression of cell viability was associated with decreased phosphorylation of known PIM kinase targets such as the eukaryotic elongation factor 4E-BP1, pro-apoptotic protein BAD, and MYC itself.

Based on these findings, we reasoned that simultaneous targeting of STAT5-induced anti-apoptotic and growth-promoting pathways may mimic the effects of STAT5 silencing inducing a more effective suppression of Ph+ ALL cell growth *ex vivo* and in mice.

Indeed, combined treatment of Ph+ ALL cells with the pan-BCL-2 antagonist Sabutoclax and the PIM kinase inhibitor AZD1208 suppressed colony formation and leukemogenesis in immunodeficient mice more effectively than either treatment alone.

In summary, this work supports the essential role of STAT5 and STAT5-regulated pathways for maintenance of Ph+ ALL cell growth. Most of the studies were based on genetic silencing of STAT5 or modulation of STAT5-regulated pathways in leukemic cells to suppress BCR-ABL1-driven STAT5 activity. However, pharmacological inhibition of STAT5 and STAT5-regulated pathways was also achieved *ex vivo* in cytokine-supplemented cell cultures that may also promote STAT5 activation independently of BCR-ABL1. Given that inhibition of STAT5 activity and suppression of Ph+ ALL cell growth were also achieved in these conditions, STAT5 inhibitors may also disrupt microenvironment-dependent activation of STAT5 in leukemic cells, possibly bypassing cell-autonomous mechanisms of resistance to TKIs.

Our study supports the importance of further development of STAT5 inhibitors for a therapy for BCR-ABL1-driven malignancies, especially at the stage of TKI-resistant disease. However, until effective STAT5 inhibitors are brought to the clinic, the simultaneous targeting of STAT5-dependent growth-promoting and anti-apoptotic pathway may represent an alternative, while other strategies may be also be investigated in parallel.

For example, increased expression of BIM was observed in STAT5-silenced Ph+ ALL cells and BIM silencing alone was sufficient to suppress the apoptosis of these cells. BIM-mimetics [6] or drugs that enhance BIM activity by blocking its binding to MCL-1 antagonists [7] may also provide an alternative approach for the treatment of TKI-resistant Ph+ ALL.

STAT5 is frequently activated in a cytokine-independent manner in Ph-like ALL due to mutations/translocations of genes such as JAK2, CRLF2, ABL1, CSF1R, and PDGFRB [8, 9]. Therefore targeting STAT5 or its downstream effectors may be beneficial for this subset of patients as well.
